# Tumor necrosis factor alpha-308 gene polymorphism and pseudoexfoliation glaucoma

**Published:** 2008-09-30

**Authors:** Oya Tekeli, M. Erol Turacli, Yonca Egin, Nejat Akar, Atilla Halil Elhan

**Affiliations:** 1 Department of Ophthalmology, Ankara University School of Medicine, Ankara, Turkey; 2 Department of Pediatric Genetics, Ankara University School of Medicine, Ankara, Turkey; 3 Department of Biostatistics, Ankara University School of Medicine, Ankara, Turkey

## Abstract

**Purpose:**

To investigate the possible association between tumor necrosis factor alpha (*TNF-α*)−308 G/A polymorphism and pseudoexfoliation (PEX) glaucoma.

**Methods:**

One hundred and ten Turkish PEX glaucoma patients and 110 healthy control subjects were enrolled in the study. All participants underwent a complete ophthalmic examination. *TNF-α*−308 was genotyped by polymerase chain reaction and restriction endonuclease analysis.

**Results:**

We found a high prevalence of the G/G genotype in PEX glaucoma patients (OR=2.88, 95% CI 1.15–7.20). The A polymorphic allele frequency was 3.2% in patients with PEX compared with 8.2% in controls (p=0.023).

**Conclusions:**

Our results suggest that *TNF-α*−308 G/A genotype is not associated with PEX glaucoma. The −308G/A variant may be a possible protective factor against PEX.

## Introduction

Glaucoma is characterized by a progressive loss of retinal ganglion cells. A previous study suggests that tumor necrosis factor alpha (TNF-α) may contribute to the disease process [1]. Pseudoexfoliation (PEX) is the most common identifiable cause of open-angle glaucoma [2]. The pathogenesis of PEX is still uncertain. PEX is associated with the excessive synthesis and deposition of an abnormal elastic microfibrillar material in all tissues of the anterior segment of the eyes and systemic tissues [3]. Excessive production and accumulation of abnormal matrix components may be due to increased de novo synthesis, decreased turnover of matrix components, or both. The extracellular matrix turnover is mediated by matrix metalloproteinases (MMPs). The tissue inhibitors of metalloproteinases (TIMPs) regulate the activity of MMPs [4]. Both MMP and TIMP expression are affected by TNF-α. An increased level of TNF-α might induce extra MMPs and/or TIMPs [5]. The *TNF-α* gene polymorphisms at position −308 and −238 are the best characterized polymorphisms and have been demonstrated to influence TNF-α expression [6].The G to A substitution at the −308 position of *TNF-α* (TNF2 allele) also results in higher TNF-α production [7]. In this study, we aimed to investigate the distribution of *TNF-α*−308 gene polymorphism in patients with PEX glaucoma.

## Methods

This case-control study included 110 patients with unilateral or bilateral PEX glaucoma, and 110 control subjects. The control group and PEX patients were selected from the General Ophthalmology Polyclinic and Glaucoma section, respectively (Department of Ophthalmology, Ankara University School of Medicine, Ankara, Turkey). On ophthalmologic examination, the anterior and posterior segments of all the cases were evaluated, and intraocular pressures (IOP) were measured with Goldmann applanation tonometry. The corneal endothelium, iris, and iris margins were evaluated for pseudoexfoliative material before dilation, and after dilation, the anterior lens surface was examined. To evaluate the angle for pseudoexfoliative material and increased pigmentation, we used gonioprisms. Criteria for inclusion in normal subjects were IOP less than 22 mmHg, normal visual fields, no evidence of glaucomatous changes in optic disc, and no history of glaucoma or ocular hypertension in first-degree relatives. These controls had no pseudoexfoliative material at the anterior lens capsule or pupillary margin. Unilateral or bilateral PEX glaucoma was diagnosed by the presence of PEX in the anterior segment, elevated intraocular pressure (>21mmHg), typical glaucomatous optic nerve changes (notching, thinner neuroretinal rim, and increased cup-to-disc ratio), and glaucomatous visual field defects. All of the patients and control subjects were Turkish. Patients and controls were informed of the experiment including a detailed explanation of all the procedures and gave their consent. The study was conducted in accordance with the principles of the Declaration of Helsinki.

### *TNF-α*−308 polymorphism

Venous blood samples were taken from all participants. Genomic DNA was isolated from peripheral blood by conventional phenol-chloroform method. The polymerase chain reaction (PCR) method was described in previous studies [8,9]. A 194 bp promoter region containing the polymorphism site was amplified [10] by using primers 5′−AAT GGA AAT AGG TTT TGA GGG T*CA T−3′ and 5′−TCT CGG TTT CTT CTC CAT CGC−3′ in which T* was not in the genomic DNA sequence and was introduced to create a potential BspHI site (New England Biolabs Inc., Ipswich, MA). PCR was performed in a total volume of 50 μl containing 5 μl of DNA template, 1 U Taq polymerase (Fermentas, Vilnius, Lithuania) in appropriate 10X buffer, 200 μM of dNTP each, 1.5 mM MgCl_2_, and 25 pM of each of the primers. Cycling conditions were as follows: initial denaturation at 94 °C for 5 min followed by 35 cycles at 94 °C denaturation for 30 s, 55 °C annealing for 30 s, and 72 °C extension for 30 s. A final extension was performed at 72 °C for 7 min. The product was electrophoresed in a 1% (wt/vol) agarose gel, and bands were visualized under ultraviolet (UV) after ethidium bromide staining.

To determine the A allele at position −308, 12.5 μl of the PCR product was digested overnight with 10 U of the enzyme, BspHI, at 37 °C and electrophoresed in a 2.5% (wt/vol) agarose gel. This digestion results in an uncut 194 bp fragment when G is present at −308 position and in two fragments of 169 bp and 25 bp when A (TNF2) is present.

### Statistical analysis

Nominal variables were evaluated by the χ^2^ test, and the odds ratio was calculated. Age was compared between groups by Student’s *t*-test. Allele frequencies were determined by gene counting. To assess the agreement between genotypes observed and those predicted by the Hardy–Weinberg equilibrium, the likelihood ratio test (G statistic) was used [11,12]. A sample size of 220 achieves 80% power to detect an effect size (W) of 0.19 using 1° of freedom χ^2^ test with a significance level (α) of 0.05. A p value less than 0.05 was considered statistically significant.

## Results

There were no statistically significant differences between the ages and sex of the patients and controls (p=0.144, p=0.589, respectively; Table 1). The distribution of the *TNF-α* G/A polymorphism was in accordance with the Hardy–Weinberg equilibrium in the PEX patients with glaucoma and the controls (p=0.657, p=0.218, respectively). The distribution of *TNF-α* G/A polymorphism between PEX glaucoma patients and controls was significantly different from each other (p=0.019). The frequency of the GG genotype in the PEX glaucoma group was higher than that of controls (OR=2.88, 95% CI 1.15–7.20; Table 2). The frequencies of the A and G alleles were significantly different between the groups (p=0.023; A=7 (3.2%), G=213 (96.8%) and A=18 (8.2%), G=202 (91.8%) for PEX glaucoma and control groups, respectively).

**Table 1:** Demographic characteristics of participants.

	**Age (years) Mean±SD**	**Female** **n (%)**	**Male** **n (%)**	**Total** **(n)**
PEX glaucoma	66.0±7.6	49 (44.5)	61 (55.5%)	110
Controls	64.2±10.1	53 (48.2)	57 (51.8)	110
p value	0.144	0.589		

**Table 2:** Genotype frequencies of the patients with PEX glaucoma and the controls.

**Genotype**	**PEX glaucoma**		**Controls**	
	**n**	**%**	**n**	**%**
GA	7	6.4	18	16.4
GG	103	93.6	92	83.6
AA	-	-	-	-
P value	0.019	0.019	0.019	0.019

## Discussion

Progressive accumulation of PEX material in the juxtacanalicular tissue causes an increase in intraocular pressure in PEX syndrome [13,14]. The pathogenesis of an aberrant matrix process is still unknown. Recent studies showed that lysil oxidase-like 1 gene polymorphisms were highly associated with PEX [15-17].

MMPs and TIMPs can play a role in the development of an abnormal extracellular matrix characteristic of PEX syndrome/glaucoma and primary open-angle glaucoma [13,14]. MMPs belongs to the endopeptidases family, and they contribute to trabecular extracellular matrix degradation [18,19]. TIMPs regulate the activity of the MMPs. An increased level of TNF-α might induce extra MMPs and/or TIMPs [5]. The functional polymorphisms in the promoter region affect plasma TNF-α levels. A G to A polymorphism at position –308 of *TNF-α* is associated with elevated TNF levels [7]. It is possible that another single nucleotide polymorphism (SNP) might be within *TNF-α* that is more relevant to TNF-α production than the *TNF-α*−308 A allele. However, a neat association between TNF-α levels and *TNF-α*−308 polymorphism exists [20]. Therefore, other polymorphisms in this gene could have less of an effect on TNF-α levels.

Lin et al. [21] evaluated the association between the *TNF-α*-308 polymorphism and primary open-angle glaucoma (POAG) in Chinese patients. They determined that the distribution of *TNF-α*−308 G/A was significantly higher in the POAG patients. Mossbock et al. [22] concluded that allelic frequencies and genotype distributions of the *TNF-α*−308G/A polymorphisms did not significantly differ between Caucasian patients with POAG and control subjects. Funayama et al [23]. did not find significant difference in the frequency of the *TNF-α*-308 G/A genotype between Japanese patients with glaucoma (POAG or normal pressure glaucoma) and healthy subjects.

In this study, we investigated the distribution of the *TNF-α*−308 polymorphism in Turkish PEX glaucoma patients. TNF G/A genotype was also found to be lower in our PEX glaucoma patients than in control subjects, we detected a high prevalence of the G/G genotype in Turkish PEX glaucoma patients. However, our main hypothesis was to demonstrate the higher frequency of the GA genotype in PEX glaucoma patients. To our knowledge, this is the first study that evaluated the association between the *TNF-α* polymorphism and PEX glaucoma. It is interesting that the *TNF-α*−308 A allele frequency is similar to previous studies from Turkey in different age groups [8,9,24]. Further, Turkish population is an admixture of several Caucasian individuals who have emigrated from different parts of the world, but there is no admixture with the Japanese and Chinese. The *TNF-α*-308 G/A polymorphism has been studied in association with different complex diseases. Although increased TNF-α expression is linked to several diseases, some studies similar to ours have reported opposite results. The exact etiology of psoriasis is still unclear but the increased levels and activity of proinflammatory cytokines, such as tumor necrosis factor (TNF)-α were suggested in the development of psoriasis [25]. However, Chunying et al. [26] performed a meta-analysis, and they found there was a protective effect for the −308G/A variant genotypes compared with the wild type homozygous genotypes. TNF-α produces hypertriglyceridemia mediated by decreased lipolysis, and increased very density lipoproteins (VLDL) secretion [27]. Parra-Rojas et al. [28] detected that homozygous GG carriers showed significantly higher serum triglycerides than GA carriers. Pereira et al. [29] performed a meta-analysis for ischemic stroke, and they showed that the G-308A polymorphism might be a protective factor against ischemic stroke in Asians only.

In our study, the GG genotype was higher in PEX glaucoma patients than in healthy controls. In conclusion, we could not find significant association between the 308 G/A genotype and the susceptibility to PEX glaucoma. *TNF-α*−308 G/G may be a genetic marker for identifying patients with increased risk of PEX glaucoma. Exactly how the GG polymorphism works to influence risk for PEX glaucoma remains unknown. Probably the genetic background of our population may be the reason for higher frequency of GG genotype in PEX glaucoma. Further studies are needed to confirm this finding.
